# Uncommonly Missed Diagnosis of Methylmalonic Acidemia (MMA) in Adults and Usefulness of Testing for MMA in Cases of Seizures/Neuropathy/Weakness/Ataxia

**DOI:** 10.7759/cureus.47577

**Published:** 2023-10-24

**Authors:** Brianna Cocuzzo, Sonia Kalirao

**Affiliations:** 1 Pediatrics, Westchester Medical Center, Valhalla, USA; 2 Neurology, Neurological Institute/Hospital Corporation of America (HCA) Healthcare, Coral Springs, USA

**Keywords:** metabolic disease, rare genetic diseases, methylmalonic acidemia, peripheral sensory neuropathy, methylmalonic acid

## Abstract

Methylmalonic acidemia (MMA) is a genetic condition affecting cobalamin metabolism causing elevated serum and urine methylmalonic acid without B12 deficiency. MMA presents with ketoacidotic hyperammonemic coma in newborns and can result in neonatal death or severe neurological disability. Rarely, this diagnosis is missed, or patients do not present until later in life. Presentation of this life-threatening condition is variable in adults. Improvement is rapid with IV cobalamin and a specialized diet. This case is intended to increase clinician’s awareness of the late presentation of this disease and the importance of high clinical suspicion and prompt diagnosis. We present a case of a 32-year-old man with seizures, polyneuropathy, ataxia, and memory loss which were unexplained until diagnosis with MMA. We aim to help clinicians understand the variable presentation and diagnostic work-up of MMA to prevent catastrophic missed diagnoses. After an extensive work-up, the patient was found to have methylmalonic acidemia and was promptly treated with high dose vitamin B12 and a specialized diet with low protein including restricted isoleucine, threonine, methionine, and valines as well as a high caloric content. The patient showed significant clinical improvement with this treatment. To our knowledge, this is the first case of MMA presenting with these symptoms in a medically stable adult. The patient was adopted from abroad and therefore, lacked access to normal newborn screenings, further complicating diagnosis. We aim to demonstrate to clinicians the importance of considering this diagnosis in patients in whom symptoms may be suggestive, particularly if they lack access to genetic or metabolic screening.

## Introduction

Methylmalonic acidemia (MMA) is an autosomal recessive genetic syndrome that is caused by mutations in the Mut- gene or other genes for cobalamin metabolism [1]. MMA typically presents in the first three days of life with pancytopenia, poor feeding, and progressive encephalopathy, and eventually coma and death [1). Several medical conditions including sepsis, hypoxic-ischemic encephalopathy, and intoxication from maternal exposure present similarly confounding clinical diagnosis. Patients often die in infancy before MMA is diagnosed [1]. Due to the early age of onset, MMA is generally considered to be a pediatric condition and many practitioners who care for adults are not familiar with it nor do they consider it in their differential diagnosis. The early onset form of the disease is associated with neonates presenting with ketoacidotic hyperammonemic coma with an extremely minimal mean survival time. Most patients diagnosed with the early onset form of the condition die within 18 months [2]. There are in fact even cases of prenatal diagnosis of MMA with homocystinuria due to inborn errors of metabolism [3]. Patients with the later onset form of the disease tend to have a longer life expectancy as well as better neurological outcomes. However, the effects of MMA, especially when entering an acute crisis, can be catastrophic at any age [2]. 

Although this condition is most often detected in children, there are reported cases of MMA presenting or being recognized for the first time in adults. Many patients with MMA diagnosed in adulthood have a history of unexplained medical problems including neurological issues that conceivably would have had better outcomes had a diagnosis been reached sooner. One such example in the literature is the case of a young woman living in India thought to have a developmental global delay involving seizures, ataxia, and dystonia who later developed episodes of acute vomiting and dehydration and renal failure by her 20s [4]. Her diagnosis was unknown, and her family was left without an explanation for her condition and eventual further deterioration for over two decades. Finally, an MRI of the brain was performed which showed T2 hyperintensities characteristic of MMA, prompting confirmatory metabolic testing [4]. The patient immediately began treatment with high-dose vitamin B12 supplementation and a low‐protein high-calorie diet and her clinical status improved with an accompanying drop in urinary methylmalonic acid excretion.

Our case, to our knowledge, is of an adult male with MMA presenting for the first time with the combination seizures, after a long seizure-free interval, peripheral polyneuropathy, and mild cognitive deficits which were slowly progressive. We hope to enlighten clinicians about the unique presentation of MMA in adults and the need to maintain a high degree of clinical suspicion when adults are presenting with unusual symptoms that could be suggestive of MMA. Although relatively uncommon in adults, a missed diagnosis of MMA can be catastrophic, and prompt recognition has the capacity to significantly reduce morbidity and mortality.

## Case presentation

**Figure 1:**
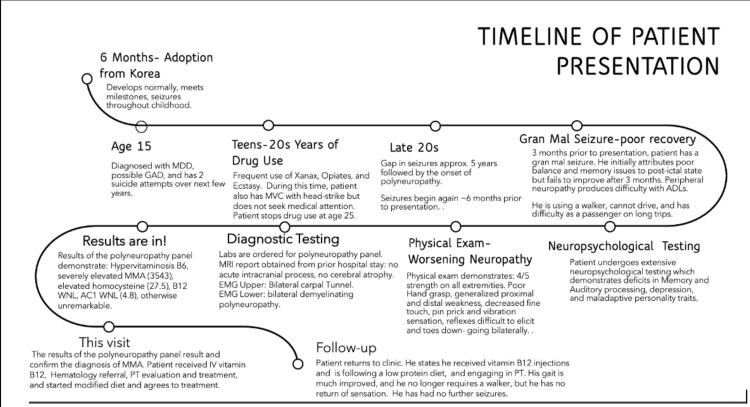
A time course of the patient's symptoms and evaluation MDD: Major depressive disorder; GAD: generalized anxiety disorder; MVC: motor vehicle accident; MMA: methylmalonic acidemia; ADLs: activities of daily living

Our patient is a 32-year-old male 15-pack-year current smoker and polysubstance (oxycodone, ecstasy, Xanax, marijuana) user adopted from Korea at six months of age with a history of childhood seizures and a few seizures as an adult initially attributed to substance withdrawal. The patient had a history of multiple hospitalizations in the past, for seizures where MRI of the brain without contrast and EEGs, all reportedly normal (reports were not available to us). The patient reportedly did have metabolic acidosis on some of these admissions or required longer stays for fluid resuscitation and medical stabilization. He also mentioned that he may have had more severe illness/altered mental status during some of these hospitalizations as he does not recall a good portion of several of them. He was on anti-epileptic medications (unsure which ones), briefly during childhood but had not been for several years prior to his presentation to the neurology clinic. He initially presented to an outpatient neurology clinic with complaints of seizures, difficulty walking, decreased sensation in the lower extremities, and progressive memory loss. At the time of his visit, he had been drug- and alcohol-free for approximately seven years. Seizure prompting presentation was described as a generalized tonic-clonic seizure lasting a few minutes without associated incontinence of bowel or bladder or tongue biting. He awoke cognitively slowed with complaints of ataxia, initially attributed to a post-ictal state. Two months after the initial seizure, he had no improvement and, therefore, presented to the neurology clinic. Further history revealed that over the past few years, but predominantly in the last few months, he developed numbness, tingling, generalized muscle cramps as well as gait ataxia requiring the use of a walker, and cognitive decline which were accelerated after the seizure. The patient's illness course is presented as a timeline in Figure 1. 

We initially considered the possibility of a genetic disorder and inquired with his adoptive parents about his family history and early childhood. Family history was unknown. Aside from the seizure disorder, he had a normal, healthy childhood and met his developmental milestones appropriately without concern regarding physical or cognitive delays. He completed high school with a 3.0 GPA and 1.5 years of college before dropping out to work in a restaurant with no reported performance issues. From age 15, he struggled with clinically diagnosed depression and suspected he suffered from anxiety and had two suicide attempts with pills and a gun. Medical history is also significant for a motor vehicle accident with a head strike, but this was after the onset of seizures.

Functionally, the patient could complete activities of daily living (ADLs) and iADLS independently without modifications, apart from limitations on mobility due to the recently developed polyneuropathy. He could not drive because of seizures. The polyneuropathy eventually progressed so he was unable to travel, even by car, for long distances, needed to hold a wall to shower, and began ambulating with a single-point cane or walker.

Neuropsychiatric evaluation, initiated at the time of these complaints, indicated deficits in his memory and auditory processing, depression, and maladaptive personality traits. The patient also reported insomnia, worse than his baseline five hours of sleep a night, and loss of appetite during this acute episode of polyneuropathy and memory loss.

A complete neurological exam revealed 4/5 strength diffusely with very poor hand grip, generalized proximal and distal weakness, decreased fine touch, pinprick, and vibration up to the knee bilaterally, decreased sensation to fine touch in both hands, difficult to elicit reflexes, and down-going Babinski bilaterally.

Given the results of his physical examination, his baseline cognitive impairment, and his reported history of progressive decline and multiple hospitalizations for seizures, cognitive deficits, ataxia, and polyneuropathy, we initiated further work-up.

Our initial differential diagnosis for a patient with complaints of seizures (with known epilepsy), polyneuropathy, and subjective memory loss, the differential diagnosis was initially broad and included diabetes, B12 deficiency, thiamine deficiency, pyridoxine deficiency, long-standing HIV infection, Lyme disease, chronic alcohol use, diabetes, thyroid disease, Charcot-Marie Tooth, amyloidosis, and multiple sclerosis among others, differential diagnosis was generated by an extensive literature review [5].

 A detailed work-up was initiated to narrow the differential diagnosis and narrow the focus of our investigation.

We obtained an MRI report from a previous hospitalization which showed no acute intracranial process and no cerebral atrophy and performed nerve conduction studies to assess the severity of the polyneuropathy. An EMG of the upper extremities showed mild bilateral carpal tunnel affecting motor components and an EMG of the lower extremities showed bilateral predominantly demyelinating peripheral neuropathy. 

Based on this presentation, the patient had a polyneuropathy panel which was demonstrated in Table 1. 

**Table 1:** A summary of key lab values for our diagnosis of methylmalonic acidemia

Vitamin B6 Level	23.7 (Elevated)
Thiamine Level	WNL
B12 Level	WNL
HgB A1C	4.8 (WNL)
Anti-Hu	Negative
Lyme Antibody	Negative
Methylmalonic Acid level	3543 (severely elevated)
Homocysteine	27.5 (elevated)

Results of laboratory investigations confirmed the diagnosis of MMA. The patient was given a referral to hematology and continues to be managed by our neurology team as well as hematology for his MMA with homocystinuria. He also participated in physical therapy and states that he feels it helped with his gait but not with the return of sensation. He can now ambulate without a cane.

Once the diagnosis of MMA was established, the patient was started on treatment with high-dose vitamin B12, initially IV, then oral, and a high-calorie low-protein diet and remains on this regimen, with good compliance and without adverse effects and is now on Keppra for seizure prophylaxis. Since initiating treatment, the patient reports significant improvement in his symptoms particularly with improved mobility, and the patient no longer requires the use of a walker and has had no further seizures but reports no return of sensation. 

## Discussion

The main strength of this case report is the clinical relevance as well as the continuity with the patient from diagnosis through treatment and clinical improvement based on our interventions. This case highlights the importance of diagnosing MMA promptly and maintaining a high index of suspicion for this condition, even in adults. For adults and children alike, in whom the diagnosis is missed, the results of metabolic crisis can be catastrophic and before a metabolic crisis, the disease can profoundly impact quality of life. Because this case is different from most existing literature, which presents MMA as a pediatric/neonatal condition, it can educate other clinicians about the unexpected presentation of MMA and the warning signs that a diagnosis was missed in an adult patient with otherwise unexplained neurological symptoms. The main limitation of this case report is the intrinsic limitation of case reports as a study design. Because this case report is focused on one individual, generalizability may be limited.

There are other cases in the literature that report on missed diagnoses of Methylmalonic acidemia with catastrophic consequences. This case is different from existing literature in that we were able to diagnose a patient with methylmalonic acidemia outside of an acute crisis and therefore, prevent some of the most catastrophic symptoms of MMA.

Overall, because missed diagnoses of MMA are rare and it is typically severe in the neonatal period, so literature is scant. However, apart from being diagnosed when the condition was less severe than other adults in the literature, his symptoms and disease progression are very similar to other reported cases of adults diagnosed with MMA later in life. One such example of the devastating impact of a missed diagnosis comes from a case of a 26-year-old man presenting for the first time with hypotonia and acute respiratory failure requiring intubation and mechanical ventilation [6]. The patient initially presented to the emergency department with a chief complaint of nausea, vomiting, and abdominal pain as well as progressive exertional dyspnea. Besides a diagnosis of renal insufficiency at age 5, the patient had been healthy, had a normal stature, and even graduated college. There were no signs of developmental delay and no family history of any genetic or metabolic diseases. Much like our patient, the signs/symptoms of the underlying disease were subtle and easily missed leading to a delay in diagnosis. Throughout this 26-year-old man’s hospital course, he neurologically deteriorated even developing quadriplegia and respiratory distress requiring mechanical intubation with several unsuccessful attempts at eventually weaning the ventilator [5]. Ultimately, a pediatrician was consulted and diagnosed the patient with hereditary MMA. As soon as the condition was identified, he was placed on a specialized diet and his neurological condition improved substantially. Methylmalonic acid levels declined substantially in just 10 days and his muscle strength substantially increased [6]. He was rehabilitated and sent home with no deficits and was maintained on a specialized diet. Had there been any further delay in the diagnosis of MMA, the patient could have succumbed to his condition. Perhaps because the disease was diagnosed when the patient’s symptoms were less severe than other cases in the literature, he had a complete remission of seizures and regained his ability to walk without assistance. While all patients in the cases we reviewed showed improvement, this near-total return to baseline is not always seen in patients with MMA. We hypothesize that this is because the patient had a recent onset of his physical symptoms and his epilepsy had not yet progressed to a level where temporal lobe sclerosis or other long-term changes were seen on MRI. It cannot be predicted from his case, what the outcome might be for a patient with more advanced disease or with substantial MRI changes representing damage to the brain parenchyma may be. There is still much research to be done to determine why patients like our 32-year-old male, and this 26-year-old male made a near-complete recovery and whether this is something that could be achieved for all patients.

This case also highlights the importance of attending to the various clinical manifestations of MMA. While the diagnosis of MMA is typically associated with metabolic encephalopathy in early infancy, the clinical presentation can be highly variable. In older children and adults, hypotonia, global developmental disability, movement disorders and dystonia, seizures, optic atrophy, psychiatric symptoms such as hallucinations, and psychosis, GI symptoms, anorexia, vomiting with ketoacidosis, constipation, pancreatitis, pancytopenia, prolonged QT, chronic renal failure, dermatitis, and hearing loss are common manifestations. In our case, symptoms such as polyneuropathy, seizures, and cognitive decline were prominent as well as history of decreased appetite. This gradual onset of a constellation of symptoms, cognitive abnormalities, and difficulty with movement parallel some of the findings demonstrated in the literature in the young lady in India discussed earlier in this report however, it seems our patient, overall, had a less severe course.

Our hope is that this case can drive future research and ensure clinician readers do not continue to miss this diagnosis and work to improve quality of life for patients with a late diagnosis of MMA. While our case is certainly unique, a review of prior literature reveals that this missed diagnosis was not a single isolated case and is something worth making clinicians aware of.

## Conclusions

Ultimately, as a patient of any age can enter a fatal metabolic crisis, or even in the absence of crisis, have a significantly reduced quality of life, we hope to advocate for routine testing for MMA when clinicians have suspicion and educate them on the presentation of this condition so future diagnoses are not missed. The blood test for methylmalonic acid is relatively inexpensive and readily available and should be included in the work-up of patients with unexplained neurological dysfunction. When a patient has some combinations of these manifestations, MMA is worth investigating. While MMA is now included in newborn screening, many patients were born outside of the United States or before it was part of the newborn screening exam, and as with any test, the newborn screen, in rare instances, can have false negatives. This case report on MMA should help clinicians understand the unique clinical presentation of MMA in adults. We especially aim to put a focus on the recognition of this medical emergency despite several confounding elements of this patient’s presentation. Clinicians must always remain alert to manifestations of serious disease, especially in medically complex patients. After reading this case, clinicians should have a framework for diagnosis and treatment of MMA and an understanding of the variability of the clinical presentation of MMA.
